# A randomized trial comparing digital and live lecture formats [ISRCTN40455708

**DOI:** 10.1186/1472-6920-4-27

**Published:** 2004-11-29

**Authors:** David J Solomon, Gary S Ferenchick, Heather S Laird-Fick, Kevin Kavanaugh

**Affiliations:** 1 Office of Medical Education Research and Development and the Department of Medicine, Michigan State University, East Lansing, MI, USA; 2 Department of Medicine, Michigan State University, East Lansing, MI, USA; 3 Department of Medicine, Michigan State University, East Lansing, MI, USA; 4 MSU-Kalamazoo Center for Medical Studies (MSU-KCMS), Michigan State University, Kalamazoo, MI, USA

## Abstract

**Background:**

Medical education is increasingly being conducted in community-based teaching sites at diverse locations, making it difficult to provide a consistent curriculum. We conducted a randomized trial to assess whether students who viewed digital lectures would perform as well on a measure of cognitive knowledge as students who viewed live lectures. Students' perceptions of the digital lecture format and their opinion as whether a digital lecture format could serve as an adequate replacement for live lectures was also assessed.

**Methods:**

Students were randomized to either attend a lecture series at our main campus or view digital versions of the same lectures at community-based teaching sites. Both groups completed the same examination based on the lectures, and the group viewing the digital lectures completed a feedback form on the digital format.

**Results:**

There were no differences in performance as measured by means or average rank. Despite technical problems, the students who viewed the digital lectures overwhelmingly felt the digital lectures could replace live lectures.

**Conclusions:**

This study provides preliminary evidence digital lectures can be a viable alternative to live lectures as a means of delivering didactic presentations in a community-based setting.

## Background

Medical education is increasingly being conducted in community-based teaching sites outside of the traditional academic medical setting [1], At the same time, the economics of health care are requiring academic physicians to be more productive[2]. These trends in academic medicine are making it more difficult to provide students and residents with consistent, high quality instruction.

Our institution has a community integrated structure where medical students spend the clinical portion of their training in one of six community campuses spread throughout the State of Michigan. Although this structure has many advantages, it is difficult to provide a consistent educational experience for the students. To help address this challenge, we implemented an all-day lecture series held at one of the community campuses two weeks before the end of the internal medicine clerkship.

The students and faculty presenters from other campuses traveled from their home campus to the campus hosting the lecture series. End-of-clerkship feedback from the students has indicated the lecture series is both valuable and well received. Traveling to the host community, however, was inconvenient and time consuming for both students and faculty presenters. In addition, it is not practical for students at our rural medicine campus to attend due to the distance (approximately 400 miles) from the other communities.

There is evidence that delivering the audio from a lecture in combination with the presenter's slides can be an effective means of delivering lectures at remote sites, and may even be as effective as traditional lectures[3,4]. We saw this as a potential solution for providing a consistent didactic curriculum in our clerkship. With the availability of inexpensive, high quality digital camcorders and software for merging audiovisual material with presentation slides, we felt including video of the presenter as well as audio from a live lecture in combination with the presenter's slides might result in a more engaging and hence more effective presentation than audio alone. During the 2003–2004 academic year, we conducted a randomized trial comparing attending the lecture series with viewing a CD-ROM-based multimedia version of the same lecture series. If the digital lectures could help students master the material at the same or similar level of understanding as live lectures, they could potentially replace the live lectures and thereby save the time lost to travel for both the students and faculty presenters. Additionally, the digital lectures would provide the same instructional opportunities for students in our rural medicine program as students in our other campuses and provide all our students the opportunity to view the presentations at their convenience.

## Methods

Students taking the third-year required internal medicine clerkship during the 2003–2004 academic year at our institution were offered the opportunity to participate in the study. Those agreeing to participate were randomized into to one of two arms of the study. The random assignment of students to the two arms of the study was done within each community to control for the potential of community differences. The control group traveled to the host community campus and attended the live lectures with their colleagues who chose not to participate in the study. The experimental group stayed at their home campus on the same day and completed a parallel set of CD-ROM-based multimedia modules made from digital recordings of the previous year's lectures. They completed these digital lectures in computer laboratories in either the community campus office or within one of the teaching hospitals.

The series included six lectures covering asthma, coronary artery disease (CAD), acute renal failure, liver disease, thyroid disease, and antibiotic pharmacology. The Clerkship Education Committee chose these topics based on their perceived importance and consistency with the Society for General Internal Medicine/Clerkship Directors of Internal Medicine (SGIM/CDIM) Curriculum Guide[5].

Between the 2002–2003 academic year when the lectures were taped and the 2003–2004 academic year when the study was conducted, the clerkship faculty decided to revise the lecture series to be more case-based though the topics were kept the same. Two of the lectures, CAD and renal failure, were not modified and kept as consistent as possible with the previous year in order to conduct the study. Though there might have been minor inconsistencies between the digital and live versions of these two lectures, the same faculty member presented each lecture during both the 2002–2003 academic year when the lectures were taped and the 2003–2004 academic year when presented live. The two lecturers also attempted to keep the live lectures as consistent as possible with the digital lectures and used the same slides that they had used the previous year. While the format of the other four lectures changed, the material covered and instructional objectives remained consistent.

At the end of the live lecture series, students were asked to complete a short examination that included four to five questions based on each of the six lectures. These questions were written by the presenters of the lectures and designed to assess student mastery of the lectures' key objectives. The students were informed that the purpose of the examination was to provide them with feedback on the mastery of the material and the presenters with feedback on the effectiveness of the lectures, and would not impact on their clerkship grade. After the students completed the exam, they were given a copy that included the correct answers and a short explanation for the correct answer. The exam forms contained no student identifiers, but students in the control group were asked to indicate on the examination form that they had agreed to participate in the study so they could be differentiated from the students who had chosen not to participate in the study.

Students in the experimental arm of the study completed the same examination in their home community after they had completed the digital lectures. They were also asked to complete a short feedback form asking whether they had any technical problems using the modules, to rate their agreement with what the researchers felt to be three potential advantages and three potential disadvantages of the modules, and whether they felt the modules could serve as a suitable replacement for live lectures.

The specific questions are listed below.

Advantages of the Modules

• Convenience of viewing the presentations when you choose.

• Avoiding having to travel to another community for an all day lecture series.

• Ability to keep copies of these presentations for use in the future.

Disadvantages of these modules

• Inability to ask questions of the presenter

• Lack of group interaction/discussion of a topic

• Just not like being in the room with the presenter

The CD-ROM modules were created using a technique developed by the first author. A manual outlining how to develop these modules is available from . They included digitized video and audio from the taped presentation inserted as a window in the PowerPoint^® ^slides from the presentation. As students displayed each of the slides, they were able to observe the presenter in the multimedia window discussing the slide that was being viewed.

Nine of the items on the exam focused on the material in the CAD and acute renal failure lectures, where the lecturers presented the lectures in the same format as they had used in the previous year when the lectures were taped. The remaining 20 examination items covered material in the other four lectures. Group differences were tested for statistical significance by both an independent sample t-test for means and a Mann-Whitney test for ranks. A Levene's test for equality of variance between the two groups was also performed. These analyses were conducted separately for the subset of items covering the material in the CAD/acute renal failure lectures and the other four lectures. A power analysis was conducted to assess the magnitude of the difference between the groups that would likely be detectable given the number of students participating in the study. The coefficient alpha reliability of the exam was also estimated. The data were presented descriptively using means, standard deviations and mean ranks within the control and experimental groups. All analyses were conducted using the Statistical Package for the Social Sciences version 11. Approval for the project was obtained from the University Committee for Research Involving Human Subjects within our institution.

## Results

A total of 96 students completed the internal medicine clerkship during the 2003–2004 academic year. As described below 56 of the students were eligible to participate in the study. A total of 29 students or 52% of the eligible students agreed to participate in the study. Complete data were available for 12 students who attended the live lectures and 17 students who completed the digital lectures.

During the first rotation, there were some technical problems in a demonstration of the digital lectures. The net result was that very few students chose to participate during that rotation. During the second and third rotations, approximately two-thirds of the students agreed to participate. There were also 20 students who were ineligible to participate. These included six students from the rural medicine program at Marquette who do not participate in the live lecture series due to the distance from the other communities. Additionally, some of the communities conducted a fourth rotation of the internal medicine clerkship due to space limitations in the three regular rotations and a live version of the lecture series was not given for the students in the fourth rotation. These 20 students all completed the CD-ROM modules but because they could not be randomized between the live and digital formats, they were not able to participate in the study.

Differences in the sample sizes for the two groups were due to some of the students in the live lecture group failing to mark that they were participating the study. During the second clerkship rotation, the proctor inadvertently failed to remind the students participating in the study to mark this information on their examinations when the exams were handed out.

A power analysis indicated that with the number of subjects in the study, it would be possible to detect differences of nine tenths of a standard deviation with a power of 80%, p < 0.05 for a one-tailed t-test.

Table 1 displays the mean, standard deviation, and average rank of the exam score for the control and experimental groups for the two sets of items. The differences between the groups for both sets of items were not statistically significant for means (t-test) or medians (Mann-Whitney) at the p < 0.05 level.

**Table 1:** Performance on the examination: CD-ROM versus live lecture format

		**Mean**	**SD**	**No.**	**p-value**
**Items from CAD and renal failure (lecture format the same for each group)**	**Live lecture**	4.42	1.08	12	
	**CD-ROM**	4.88	2.00	17	
	**t-test^†^**				0.22 (one-tailed)
	**Mann Whitney U**				0.56 (exact test)
	**Levene's Test for equality of variances**				0.026
**Items from other four lectures (lecture format differed for control & treatment groups)**	**Live lecture**	9.25	3.11	12	
	**CD-ROM**	9.00	2.72	17	
	**t-test^†^**				0.41 (one-tailed)
	**Mann Whitney U**				0.91 (exact test)
	**Levene's Test for equality of variances**				0.96

^†^The t-test was calculated based on unequal variances. Differences in variances were tested via a Levine test and found to be statistically different in the two groups.

^‡^The t-test was calculated based on equal variances. Differences in variances were tested via a Levine test and found not to be statistically different in the two groups.

The Levene's test for equality of variance between the control and experimental groups was statistically significant (p = 0.03) for the CAD and renal failure items. The variation among the scores of students who observed the CD-ROMs was almost twice as large as for students who observed the live lectures. There was no statistically significant difference among the groups for the variance of the items from the other four lectures.

The coefficient alpha reliability for the items covering CAD/renal failure and the items covering the other four lectures were 0.33 and 0.66 for the 9 and 20 item scales respectively and was 0.70 for the combined 29-item exam.

The 17 students who completed the digital lectures also completed a short feedback form on their experiences and impressions of the digital lecture format. These data are presented in Table 2.

**Table 2:** Feedback on the CD-ROM Based Lectures

	**Yes**	**No**		
Did you have any technical difficulties viewing the modules?	16 (94.1%)	1 (5.9%)		
**Advantages of the Modules**	**Very Important**	**Important**	**Slightly Important**	**Not Important**
Convenience of viewing the presentations when you choose.	12 (70.6%)	5 (29.4%)	0 (0.0%)	0 (0.0%)
Avoiding having to travel to another community for an all day lecture series.	15 (88.2%)	2 (11.8%)	0 (0.0%)	0 (0.0%)
Ability to keep copies of these presentations for use in the future.	9 (52.9%)	3 (17.6%)	5 (29.4%)	0 (0.0%)
**Disadvantages of these modules**				
Inability to ask questions of the presenter	3 (17.6%	6 (35.3%)	6 (35.3%)	2 (11.8%)
Lack of group interaction/discussion of a topic	3 (17.6%	5 (29.4%)	1 (5.9%)	8 (47.1%)
Just not like being in the room with the presenter	0 (0.0%)	1 (5.9%)	5 (29.4%)	11 (64.7%)
	**Strongly Agree**	**Agree**	**Disagree**	**Strongly Disagree**
**These modules can serve as an adequate replacement for the all day Crush the Boards lecture series.**	**10 (58.8%)**	**6 (35.3%)**	**0 (0.0%)**	**0 (0.0%)**

## Discussion

There were no statistically significant differences found between students who viewed the live and CD-ROM based lectures. The observed mean scores in the two groups were in fact almost identical. Unfortunately, the small sample size limits the power of the study and confidence in which we can assert that digital lectures can be as effective as live lectures in increasing students' knowledge. The study does suggest that it is unlikely that there are large differences in the performance of students who view CD-ROM based lectures as opposed to live lectures and adds to the growing body of literature concerning the effectiveness of technology for implementing distance learning in medical education.

There was a statistically significant difference in variances among the two groups for the items covering the CAD/renal failure lectures. The standard deviation in the scores was twice as large for the students who completed the digital modules. The differences in the dispersion are also evident in the range of values in each group. It is not clear why there was more variation in the scores among the students who completed the digital modules. It may be related to the technical problems encountered by many of the students in accessing the modules, though one would expect this would have resulting in extending the lower tail of the distribution but not the upper tail. It may have also in part reflected the impact of discussions that occurred during the live lectures that may have reduced the variability among the students in their responses to the examination.

Despite the fact that almost all of the students experienced some technical difficulties using the modules, they all agreed and most strongly agreed the modules could serve as an adequate replacement for live lectures. They were particularly appreciative of not having to travel to another community to attend didactic presentations and having the flexibility of viewing the modules at their convenience. Of the three potential disadvantages of the format that were listed on the feedback form, they felt their inability to ask a question of the presenter was the most important. In the future we are considering using a web-based bulletin board system as a means of allowing students to ask questions of the presenter.

The number of students with technical difficulties viewing the modules was surprising. We had tested the modules on a variety of different computers with very few problems. In a few cases, the CD-ROMs we distributed apparently had not been copied correctly. Additionally, we switched the video formats from MPEG to Windows media files. We assumed there would be less compatibility problems with the Windows media files given that this is a format developed by Microsoft. Unfortunately, we later found out the Windows media files require software that was not shipped with earlier versions of Windows. We expect this was a significant cause of the technical problems the students experienced.

We are now using a commercial software package which greatly simplifies the process of creating the digital lectures and allows them to be distributed over the Web as well as via CD-ROM requires no special software minimizing the compatibility issues. Students who completed the fourth rotation of the clerkship and did not participate in the study were provided with the new version of the modules. Only one of the 12 students indicated they had technical problems accessing the lectures off the CD-ROM disks on which the modules were distributed and that student was able to access the modules via the Web. Such combined Web and CD-ROM distance learning formats have been shown to be effective in a number of educational settings [6,7].

## Limitations

There were several important limitations in the study. First is the very small sample size which limited the power of the study for detecting differences in the performance of the students completing the live and CD-ROM based lectures. It also increased the likelihood the two groups of students were not equivalent. Since there were no student identifiers on the exams, it was not possible to compare the characteristics of the two groups. The outcome measure was a locally developed test. While the items were written by the presenters and based on the major objectives in their lectures, there was no assessment of validity other than content validity. It is also not clear the extent the findings of this study can be generalized to other digital lecture formats.

## Conclusions

Although the data collected in this study were limited, it provides some evidence that digital lectures are both well received by students and can provide a satisfactory substitute for live lectures from a performance standpoint.

## Competing interests

The author(s) declare that they have no competing interests.

## Authors' contributions

DJS designed the study, developed the feedback instrument on for the digital modules conducted the statistical analyses and wrote the first draft of the paper. GSF, HSF and KK developed and gave presentations, wrote questions for the knowledge examination and edited and helped revise the manuscript.

## Pre-publication history

The pre-publication history for this paper can be accessed here:


